# Serum MiRNA signatures in insulin resistance: toward personalized diabetes prediction

**DOI:** 10.1097/MS9.0000000000004910

**Published:** 2026-04-02

**Authors:** Hasibullah Aminpoor, Muhammad Zaib, Muhammad Khizar, Muhammad Bilal, Hasiba Karimi

**Affiliations:** a Faculty of Medicine, Kabul University of Medical Sciences “Abu Ali Ibn Sina”, Kabul, Afghanistan; b Faculty of Medicine, Manifest Medical Research Co, Georgian American University, Tbilisi, Georgia; c Faculty of Medicine, Shahida Islam Medical and Dental College, Lodhran, Punjab, Pakistan; d Faculty of Medicine, Bezmialem Vakif University, Istanbul, Turkey

**Keywords:** biomarkers, gene regulation, insulin resistance, metabolic disease, microRNAs, personalized medicine, type 2 diabetes mellitus

## Abstract

The rising prevalence of insulin resistance and its progression to Type 2 Diabetes Mellitus (T2DM) necessitates effective early-prediction strategies. Circulating microRNAs (miRNAs) modulate key insulin-signaling pathways and may serve as non-invasive biomarkers for metabolic dysfunction. MiRNA signatures, such as miR-103/107, miR-126-5p, miR-181b, miR-29a-3p, and miR-375-3p, have been associated with insulin resistance in populations from Europe, the Middle East, and South Asia. Integrating mechanistic insight, diagnostic application, and multi-miRNA network analysis advances the field toward personalized diabetes prediction. However, current research is limited by small cohorts, heterogeneous methodologies, crosssectional designs, and ethnic underrepresentation. Translating miRNA profiling into clinical practice requires standardized protocols, multinational longitudinal studies, and interventional validation. Such approaches could enable targeted preventive interventions and inform precision medicine strategies in insulin-resistant populations. Current evidence remains exploratory and should be interpreted cautiously until validated in large-scale, longitudinal studies .

*To the Editor*,

The global prevalence of insulin resistance and its progression to type 2 diabetes mellitus (T2DM) represents a major public health challenge. Early identification of at-risk individuals is critical for timely preventive interventions. In this context, circulating microRNAs (miRNAs) have emerged as promising non-invasive biomarkers that could enable personalized diabetes prediction, bridging molecular pathophysiology with clinical practice.

While the potential of miRNA panels is promising, current evidence remains exploratory, and large-scale, longitudinal validation studies are needed before clinical implementation.

Insulin resistance arises from impaired insulin-mediated glucose disposal in skeletal muscle, adipose tissue, and liver, combined with beta-cell dysfunction and compensatory hyperinsulinaemia. MiRNAs (small non-coding RNAs of ~19–24 nucleotides) regulate multiple mRNAs encoding key insulin-signaling proteins, including insulin receptor substrates (IRS), PI3K subunits, and GLUT4 transporters^[^1,2^]^. For instance, miR-103/107 downregulates caveolin-1, reducing insulin sensitivity in experimental models^[^3^]^. The stability of circulating miRNAs within extracellular vesicles or bound to AGO2 makes them attractive candidates for non-invasive risk assessment^[^2,3^]^.

Clinical studies across diverse populations have validated the relevance of serum miRNAs in insulin resistance. In Iran, miR-181b and miR-126-5p were progressively reduced in pre-diabetic and T2DM individuals compared with healthy controls^[^4^]^. Sri Lankan cohorts demonstrated upregulation of miR-29a-3p and miR-375-3p in T2DM, with careful validation of stable reference miRNAs^[^5^]^.

However, most studies are small, largely cross-sectional, and show substantial population heterogeneity, limiting generalizability^[^2,4–6^]^. Normalization strategies, differences between serum and plasma, and RNA isolation methods remain major sources of methodological variability^[^5^]^. These limitations underscore that current miRNA signatures are not yet ready for routine clinical prediction and require cautious interpretation .

Integrating such panels with established indices, including HOMA-IR and adiponectin/leptin ratios, may in the future guide earlier interventions in high-risk populations, including individuals in the United States, India, and sub-Saharan Africa^[^2,6^]^. MiRNA profiling also elucidates mechanistic pathways of insulin resistance, providing translational insights for clinical application.

Innovations are extending beyond association studies. Mechanistic work in skeletal muscle demonstrates that miRNAs mediate insulin signaling attenuation via JNK/NF-κB activation and mitochondrial substrate dysregulation^[^7^]^. Systems biology and bioinformatics enable multi-miRNA network analyses, generating predictive signatures rather than relying on single molecules^[^3^]^. Research in Bahrain and China is expanding these approaches to ethnically diverse populations, reflecting global variations in insulin resistance^[^6^]^.

Nevertheless, implementation in clinical practice is still premature. Validation in multi-ethnic, longitudinal cohorts is essential, as are interventional studies to determine whether miRNA-guided strategies improve outcomes. Only after such work can miRNA panels be incorporated into evidence-based preventive or therapeutic decision-making.

In conclusion, Serum miRNA profiling represents a promising avenue for personalized prediction of insulin resistance and T2DM, but current evidence is exploratory. Future research should prioritize: (1) standardized sample collection and RNA processing; (2) multi-center, longitudinal validation in diverse populations; (3) assessment of clinical utility in risk stratification and intervention response. Addressing these steps will be crucial before miRNA panels can transition from research tools to clinically actionable biomarkers.

The authors affirm adherence to transparency and ethical guidelines, including the TITAN guideline for reporting Artificial Intelligence and associated analyses^[^8^]^.

## Data Availability

Not applicable.
